# Systemic treatment of advanced non-small cell lung cancer: controversies and perspectives

**DOI:** 10.1007/s12254-018-0408-y

**Published:** 2018-05-18

**Authors:** Andreas Tiefenbacher, Robert Pirker

**Affiliations:** Department of Medicine I, Medical University of Vienna, 1090 Vienna, Austria

**Keywords:** Immune checkpoint inhibitors, Palliative chemotherapy, Targeted therapies, Value-based judgements

## Abstract

Patients with advanced non-small cell lung cancer receive first-line therapy with chemotherapy, targeted therapies in case of tumors with driver mutations, and more recently also immune checkpoint inhibitors. Important controversies include the role of targeted therapies in combination with chemotherapy, optimal sequencing of treatments, treatment guidance by means of predictive biomarkers, and value-based judgements of treatments.

Patients with advanced non-small cell lung cancer (NSCLC) are currently treated with chemotherapy, targeted therapies, and immune checkpoint inhibitors. Treatment depends on patient characteristics (e.g., age, performance status, comorbidity, pretreatment status), tumor histology and molecular tumor characteristics. Besides these factors, access to and affordability of anticancer drugs are of increasing importance, particularly in middle and low income countries.

## Palliative chemotherapy

Palliative chemotherapy improves overall survival and relieves cancer-related symptoms, and will remain standard treatment for most patients in the future. First-line chemotherapy is currently standard for patients without documented driver mutations in their tumors and for those with low programmed death-ligand 1 (PD-L1) levels. Patients with documented driver mutations and those with high PD-L1 levels receive first-line therapy with tyrosine kinase inhibitors and pembrolizumab, respectively, and many of these patients will also receive chemotherapy later during the course of their disease. Maintenance chemotherapy and second-line chemotherapy will also remain standard for selected patients in the future.

The type of platin, carboplatin or cisplatin, remains a matter of some debate. The more widely use of carboplatin in some countries is mainly driven by its less time-consuming administration compared to the one of cisplatin. Nedaplatin, a platin with less nephrotoxicity and gastrointestinal toxicity, might gain importance in the future [1]. Customized chemotherapy based on molecular biomarkers faces many challenges due to the molecular complexity of NSCLC and will remain experimental in the foreseeable future.

## Palliative chemotherapy plus targeted therapies

Angiogenesis inhibitors added to palliative chemotherapy have been established as standard therapies. Bevacizumab added to first-line chemotherapy improved outcome, although a survival benefit was proven only for carboplatin plus paclitaxel as chemotherapy backbone [2]. Thus bevacizumab is more commonly used with carboplatin plus paclitaxel than with cisplatin-based protocols. When added to second-line treatment with docetaxel, ramucirumab and nintedanib improved overall survival of patients with advanced NSCLC and those with adenocarcinomas, respectively [3, 4]. Some controversy, however, exists whether the survival benefits achieved with these combined treatments compared to docetaxel alone are clinically meaningful.

Epidermal growth factor receptor (EGFR) directed monoclonal antibodies (cetuximab, necitumumab) added to first-line chemotherapy improved survival of patients [5–7]. Although these antibodies led to similar survival benefits, only necitumumab has been approved in combination with platin plus gemcitabine for patients with advanced squamous cell NSCLC. High EGFR expression and EGFR fluorescence *in situ* hybridization (FISH) positivity were shown to predict those patients who derive the greatest benefit from the addition of cetuximab or necitumumab to first-line chemotherapy [8, 9]. In our opinion, therefore, either of these markers lends itself for selection or at least enrichment of patients who will derive a clinically meaningful survival benefit from either of these antibodies [10]. Necitumumab has been approved by the European Medicines Agency for treating advanced stages of squamous NSCLC with positive expression of EGFR on the cancer cell surface, independent of the degree of positive expression.

## Targeted therapies in advanced driver-mutation-positive NSCLC

EGFR tyrosine kinase inhibitors (TKIs) have been established as first-line therapy in patients with advanced EGFR mutation-positive NSCLC (for review see ref. [11]). After a median duration of 8–13 months, however, patients develop drug resistance which is due to the T790M resistance mutation in approximately 50% of these patients. Third-generation EGFR TKIs, target EGFR mutations and the T790M resistance mutation but spare wild-type EGFR, and, therefore, should be more active and less toxic than first- or second-generation TKIs [12]. Osimertinib resulted in superior progression-free survival and overall survival compared to chemotherapy in patients who had acquired T790M-mediated resistance and, therefore, has become standard treatment in patients with T790M-mediated resistance [13]. Recently, osimertinib increased progression-free survival compared to erlotinib or gefitinib in the first-line treatment of patients with advanced EGFR mutation-positive NSCLC and survival data are pending [14]. This raises the question of the optimal best sequencing of treatments, and, in particular, whether osimertinib should become the new standard for first-line treatment of patients with advanced EGFR mutation-positive NSCLC [15].

Other strategies to improve outcome have also been studied [16]. The combination of erlotinib with bevacizumab was promising but these results require confirmation in a phase 3 trial [17]. The clinical value of immune checkpoint inhibitors in patients with advanced EGFR mutation-positive NSCLC remains a matter of debate because they may have less active against tumors with driver mutations and, when combined with TKIs, may increase toxicity, in particular pulmonary toxicity.

Several ALK inhibitors (crizotinib, ceritinib, alectinib, brigatinib, and lorlatinib) have shown efficacy in patients with ALK-positive tumors and some of them have already been approved, either as first-line treatment or as later lines of treatment [18, 19]. The optimal sequencing of these various drugs is increasingly becoming a matter of debate [18, 19]. Patients with ROS1-positive NSCLC are treated with crizotinib and those with BRAF-V600 mutation-positive advanced or metastatic NSCLC are treated with a combination of dabrafenib and trametinib.

## Immune checkpoint inhibitors

Immune checkpoint inhibitors have improved survival compared to docetaxel in patients with advanced NSCLC who have been pretreated with chemotherapy [20–23]. Pembrolizumab increased survival compared to chemotherapy in treatment-naive patients with advanced NSCLC and PD-L1 expression in 50% or more of tumor cells, while nivolumab failed to improve survival [24, 25]. Reasons for these discrepant findings remain unclear.

Patient selection by predictive biomarkers remains controversial. Increasing PD-L1 levels have been associated with increasing benefit from these drugs [21]. Mutational tumor burden appears to be another potential biomarker [26]. Patients receiving immune checkpoint inhibitors as first-line therapy will switch to chemotherapy at the time of disease progression. However, little is known whether pretreatment with immune checkpoint inhibitors impacts on the outcome of subsequent chemotherapy.

## Novel clinical trial designs

Novel trial designs aim at speeding up the clinical development of anticancer drugs. One strategy focusses on early but conditional approval of drugs, subsequent drug monitoring in the real-world setting, and corresponding adaption of the approval. The second strategy focusses on master protocols which allow simultaneous evaluation of several agents. Drugs with promising efficacy will be further evaluated, while those with insufficient efficacy will be dropped early on. The overall including long-term impact of both strategies remains to be seen.

## Value-based judgments

Increasing costs of modern anticancer drugs have stimulated the discussion on drug values. Value-based judgements of anticancer drugs balance the magnitude of clinical benefit against costs. The ESMO-Magnitude of Clinical Benefit Scale is a standardized, generic, validated tool to assess the magnitude of clinical benefit that can be expected form anticancer therapies [27]. The incremental cost–effectiveness ratio (ICER) is often used to evaluate the value of a new anticancer drug. ICER refers to the costs per life year gained or costs per quality-adjusted life year gained. A drug is considered cost-effective if its ICER is below a certain threshold which depends on the country and may range from about 20,000 to more than 50,000 euros.

The evaluation of the clinical benefit of anticancer therapies is a complex and rapidly moving field and its long-term impact on guiding treatments remains to be seen. Despite all these developments, knowledge and clinical experience of physicians will remain of paramount importance for optimal care of patients with lung cancer.
